# Chinese hamster ovary cell line DXB-11: chromosomal instability and karyotype heterogeneity

**DOI:** 10.1186/s13039-021-00528-3

**Published:** 2021-02-17

**Authors:** Victoria I. Turilova, Tatyana S. Goryachaya, Tatiana K. Yakovleva

**Affiliations:** 1 Laboratory of Cell Morphology, Institute of Cytology, Russian Academy of Sciences, Tikhoretsky ave., 4, St Petersburg, Russia 194064; 2 Centre of Cell Technologies, Institute of Cytology, Russian Academy of Sciences, Tikhoretsky ave., 4, St Petersburg, Russia 194064

**Keywords:** CHO DXB-11 cell line karyotype, Chromosomal instability, Karyotype heterogeneity, Chinese hamster ovary cells, CHO chromosomes

## Abstract

**Background:**

Chinese hamster ovary cell lines, also known as CHO cells, represent a large family of related, yet quite different, cell lines which are metabolic mutants derived from the original cell line, CHO-ori. Dihydrofolate reductase-deficient DXB-11 cell line, one of the first CHO derivatives, serves as the host cell line for the production of therapeutic proteins. It is generally assumed that DXB-11 is identical to DUKX or CHO-DUK cell lines, but, to our knowledge, DXB-11 karyotype has not been described yet.

**Results:**

Using differential staining approaches (G-, C-banding and Ag-staining), we presented DXB-11 karyotype and revealed that karyotypes of DXB-11 and CHO-DUK cells have a number of differences. Although the number of chromosomes is equal—20 in each cell line—DXB-11 has normal chromosomes of the 1st and 5th pairs as well as an intact chromosome 8. Besides, in DXB-11 line, chromosome der(Z9) includes the material of chromosomes X and 6, whereas in CHO-DUK it results from the translocation of chromosomes 1 and 6. Ag-positive nucleolar organizer regions were revealed in the long arms of chromosome del(4)(q11q12) and both chromosome 5 homologues, as well as in the short arms of chromosomes 8 and add(8)(q11). Only 19 from 112 (16.96%) DXB-11 cells display identical chromosome complement accepted as the main structural variant of karyotype. The karyotype heterogeneity of all the rest of cells (93, 83.04%) occurs due to clonal and nonclonal additional structural rearrangements of chromosomes. Estimation of the frequency of chromosome involvement in these rearrangements allowed us to reveal that chromosomes 9, der(X)t(X;3;4), del(2)(p21p23), del(2)(q11q22) /Z2, der(4) /Z7, add(6)(p11) /Z8 are the most stable, whereas mar2, probably der(10), is the most unstable chromosome. A comparative analysis of our own and literary data on CHO karyotypes allowed to designate conservative chromosomes, both normal and rearranged, that remain unchanged in different CHO cell lines, as well as variable chromosomes that determine the individuality of karyotypes of CHO derivatives.

**Conclusion:**

DXB-11and CHO-DUK cell lines differ in karyotypes. The revealed differential instability of DXB-11 chromosomes is likely not incidental and results in karyotype heterogeneity of cell population.

## Background

Chinese hamster ovary cell lines known as CHO cells represent a large family of related, but quite different cell lines which are metabolic mutants derived from the original cell line, CHO-ori [1–3], by cloning, selection or induced mutagenesis. Establishment of CHO-ori cell line which resulted from spontaneous transformation of Chinese hamster ovary cells in culture [1] can be considered as the beginning of prolonged and intricate history of these cells. Due to the fact that CHO cell specimens, often under different names, were transferred to different researchers and laboratories and cultivated in various conditions, this history is hard to trace [2, 3].

The unique plasticity of the CHO genome has made these cells the major mammalian host cells for manufacturing of protein pharmaceuticals. The most industrially relevant CHO cell lines are CHO-K1 [4], CHO-S [2], CHO-DXB11 [5], and CHO-DG44 [6].

The undoubted advantages of immortalized CHO cells include a high proliferation rate, and the ability to adapt to genetic manipulations and culture conditions. At the same time, these properties are associated with an increased mutation rate, changes in the genome structure and DNA methylation pattern [7–9]. The karyotype heterogeneity of CHO cells increases with prolonged cultivation [10] and is inevitably reproduced in course of subcloning of cells [11–13]. Genetic instability is a challenge to obtain long-lived, stable, highly productive recombinant strains, and to ensure the quality of the synthesized protein [3, 14]. For example, structural changes of the genome at the transgene integration site lead to loss of productivity of recombinant CHO-DG44 cells producing immunoglobulin G [9]. The correlation between chromosome rearrangements and production instability was also demonstrated in alkaline phosphatase secreting cell line CHO-SEAP [15]. It is assumed that instability of the producer cell strains stems from chromosomal/genomic instability of the host cell line [12]. Therefore, in recent years research has been focused on identifying the stable genome regions for targeted transgene integration.

Karyotype heterogeneity of the cell population can be studied with two main methods: conventional karyotyping (G-, Q-banding) or fluorescence in situ hybridization with chromosome-specific painting probes (multicolor FISH, M-FISH). In the first case, karyotype analysis compares unique banding patterns of chromosomes and can provide comprehensive information regarding chromosome rearrangements in individual cells although it may fail to identify all chromosomal material. On the contrary, M-FISH analysis provides complete identification of chromosomal material, but intrachromosomal rearrangements such as deletions, inversions, duplications, isochromosomes remain invisible. In addition, unlike with banding, the number of cells that can be karyotyped by M-FISH method is limited. We chose analysis of G-banded chromosomes as a simple, reliable, and low-cost method which allows to establish the type and level of chromosomal changes in individual cells, and therefore to estimate true karyotype heterogeneity. Besides, G-banded karyotype may be important for establishing the origin of recombinant strains derived from certain host cells. Finally, karyotyping of CHO cell lines—original CHO [16], DG-44 [11], CHO-DUK [12, 15]—had been performed using G-banding, which made it possible to compare our results to previously obtained data.

The DXB-11 cell line is one of the first CHO derivatives, generated at Columbia University. This line was developed as a result of chemical mutagenesis followed by gamma irradiation and represents a radiation mutant with a deletion of one allele of the dihydrofolate reductase (*DHFR*) gene and missense mutation (T137R) in the second allele [5, 17]. Double inactivation of *DHFR* made this cell line very useful for transgenesis with a functional *DHFR* gene. Further selection of recombinant cells in a medium containing methotrexate results in amplification of both *DHFR* and the gene of interest. Thus, the DXB-11 cell line is the host in relation to the producer strains obtained on its basis.

The cell line DXB-11 is known under different names, including CHO K1 DUX-B11 [18], DUKX [19], DUK-XB11 [2], CHO^dhfr−^ [20], CHO-DUK [12, 15]. These are generally believed to represent the same cell line. The G-banded and ranked chromosomes of DXB-11 [21] and DUKX cells [19] were presented by independent research groups. Already in these early works, the chromosomes specific for each cell line can be noticed despite the set of similar chromosomes in both lines.

Later, CHO^dhfr−^ [20] and CHO-DUK cells from American Type Culture Collection [12, 15] showed similarity of their karyotypes (despite the different interpretation by the authors of the structure of individual chromosomes) as well as similar composition of chromosomes as compared to DUKX cells [19]. The data on the karyotype of DXB-11 cell line could not be found, with the exception of the karyotypes of the two recombinant lines obtained on the basis of DXB-11 [22]. The genome of DXB-11 cells has been sequenced [17].

The DXB-11 cell line was delivered to the Institute of Cytology (Russian Cell Culture Collection) from Columbia University, New York, USA, in 1984. Cytogenetic analysis of these cells has not been performed until now.

Here, we describe the G-banded karyotype of DXB-11 cells and present our estimation of the instability of each chromosome. We have found out that DXB-11 karyotype differs from the karyotype of CHO-DUK cell line. Besides, assessment of frequency of chromosome participation in additional structural rearrangements (ASR) reveals differential instability of individual DXB-11 chromosomes. Comparative karyotype analysis of CHO cell lines, including CHO-DUK and our data on DXB-11, allows us to make a distinction between stable and variable CHO chromosomes.

## Material and methods

### Cell line, culture conditions and chromosome preparation

DXB-11 cell line from Collection of Cell Cultures of Vertebrates (Russian Cell Culture Collection, Institute of Cytology, St Petersburg) was examined. DXB-11 cells were adherently maintained in T-25 culture flasks (ThermoFisher Scientific, Denmark) containing 5 mL F-12К (Gibco, UK) supplemented with 10% fetal bovine serum (Gibco, UK) at 37 °C in a humidified incubator with 5% CO_2_. Cells were passaged twice a week at a ratio of 1:3 just as 90% confluence was reached. Cytogenetic analysis was performed at passage 8 over 30 days of cultivation after decryoconservation. Cells in the exponential growth phase were exposed to KaryoMAX™ Colcemide™ Solution in PBS (Gibco, USA) at a final concentration of 0.1 µg/mL for 1 h, trypsinized, washed with phosphate buffered saline (PBS), and treated with 0.075 M KCl at room temperature for 15 min. Then the cells were fixed in prefrozen (− 20 °C) methanol : acetic acid (3:1) solution three times for 20 min each. Chromosome spreads were obtained by dropping of cell suspension onto cold wet slides which were air dried after.

### G-, C-banding and AgNOR staining

Preparations of metaphase chromosomes were baked at 60 °C for 16 h and G-banded by treating with 0.02% trypsin followed by staining with 2% Giemsa solution in phosphate buffer [23]. To reveal constitutive heterochromatin, C-banding method with barium hydroxide and Giemsa staining was performed according to Sumner [24]. Silver staining indicative of nucleolar organizer regions (NORs) activity was obtained using Howell and Black technique [25].

### Chromosome analysis

The chromosomes were analyzed with Axio Scope.A1 microscope coupled to an image capturing system AxioCam Cm1 (Carl Zeiss, Germany) using a 100 × oil objective. The number of chromosomes in 100 G-banded metaphase spreads was determined. At least 35 C- and Ag-stained metaphase spreads per each technique were examined using a 100 × oil objective. Karyotyping of 112 cells was performed on Axio Imager A2 microscope (Carl Zeiss, Germany) equipped with the Ikaros4 Karyotyping System (MetaSystems, Germany) using a 63 × oil objective. Percent of polyploid cells was estimated by viewing of 1000 metaphase plates. The nomenclature for Chinese hamster (*Cricetulus griseus*) chromosomes at 325-band [26] and 575-band [27] levels of resolution was employed for chromosome identification. Karyotype and structurally rearranged chromosomes described according to the International System for Human Cytogenetic Nomenclature ISCN, 2016 [28]. The frequency of chromosome involvement in clonal and nonclonal ASR was calculated as the ratio of the number of structural rearrangements of each chromosome to the number of the corresponding chromosome in 112 karyotypes. To compare frequencies, Fisher’s exact test was used. For calculations, a *p* value < 0.05 was considered statistically significant.

## Results

### Karyotype analysis

The number of chromosomes in DXB-11 cells ranged from 18 to 22 (Table 1) while the modal chromosome number was 20 (78.00% of cells). The decrease in the number of chromosomes was a result of the appearance of dicentric chromosomes in individual cells. The increase in the number of chromosomes was due to the appearance of an additional copy of any of the following chromosomes: 5, del(2)(q11q22), mar1, mar2, or unidentified chromosomes, as well as chromosome fragmentation and the presence of short and long arms as independent chromosomes. Polyploid cells represent 9.80% of cell population.

**Table 1:** Chromosome number variation in DXB-11 cells

The number of chromosomes per cell	18	19	20	21	22
The number of cells	2	6	78	13	1

**Fig. 1:**
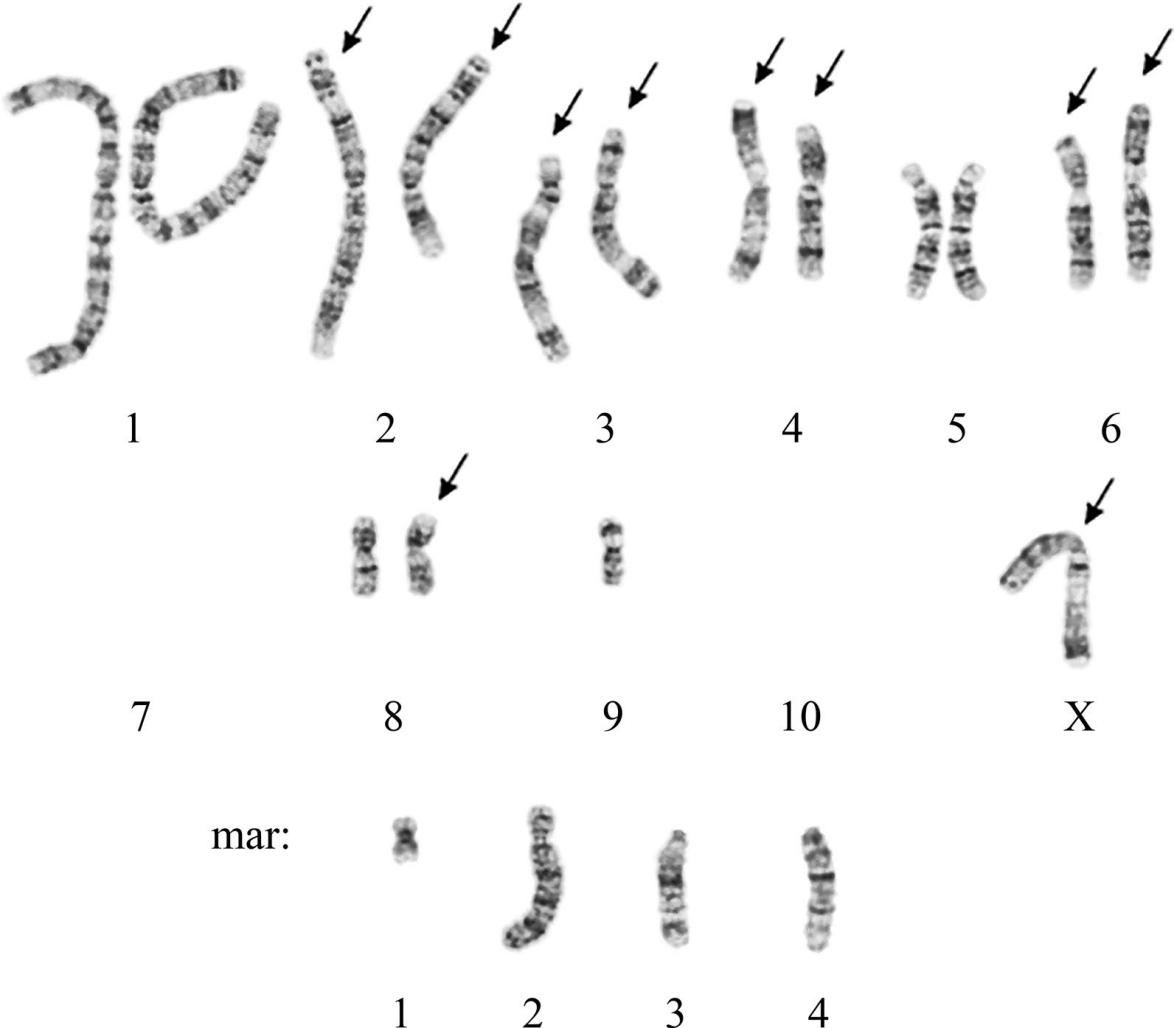
G-banded karyotype of Chinese hamster ovary DXB-11 cell line (main SVK). 20,–X,der(X)(Xpter → Xq11::3p13 → 3p21::4p11 → 4pter),del(2)(pter → p23::p21 → qter),del(2)(pter → q11::q22 → qter),inv(3)(pter → p26::q11 → p26::q11 → qter),der(3)(?8q::3p13 → 3q37::?),del(4)(pter → q11::q12 → qter),der(4)(3pter → 3p21::4p11 → 4q1?5::4q?::4q112 → 4qter),add(6)(?::p11 → qter),der(6)(Xq?::6q13 → 6p11::?::6q15 → 6qter),–7,–7,add(8)(pter → q11::?),–9,–10,–10,+4mar. The arrows indicate structurally rearranged chromosomes

In spite of limited numerical variability, DXB-11 cell line was characterized by chromosomal instability and karyotype heterogeneity. Amongst the 112 analyzed metaphase plates with 20 chromosomes, 19 cells (16.96%) had the same karyotype accepted as the main structural variant (SVK), since it most accurately reflects the combination of normal and structurally rearranged chromosomes (Fig. 1). The remaining 93 metaphase plates (83.04%) showed various clonal and nonclonal ASR. In 23 cells (20.54%) only one chromosome was affected by ASR, whereas in 70 cells (62.50%) several chromosomes were involved in ASR.

The main SVK consisted of 6 normal and 14 structurally rearranged chromosomes, including 4 marker chromosomes (Fig. 1). Among normal chromosomes, only chromosomes of the 1st and the 5th pairs were represented by both homologues, whereas chromosomes 8 and 9—by one homologue. Structural chromosome rearrangements were a complex combinations of deletions, inversions and translocations, often involving centromeric and pericentromeric loci, so it was possible to identify only certain regions of abnormal chromosomes belonging to the normal ones.

G- and C-banding revealed one rearranged X chromosome (Figs. 1, 3a). Its short arm remains in chromosome der(X)t(X;3;4), whereas the rearranged long arm is the part of chromosome der(6) (Fig. 3a). The long arm of another X chromosome homologue is likely absent.

**Fig. 2:**
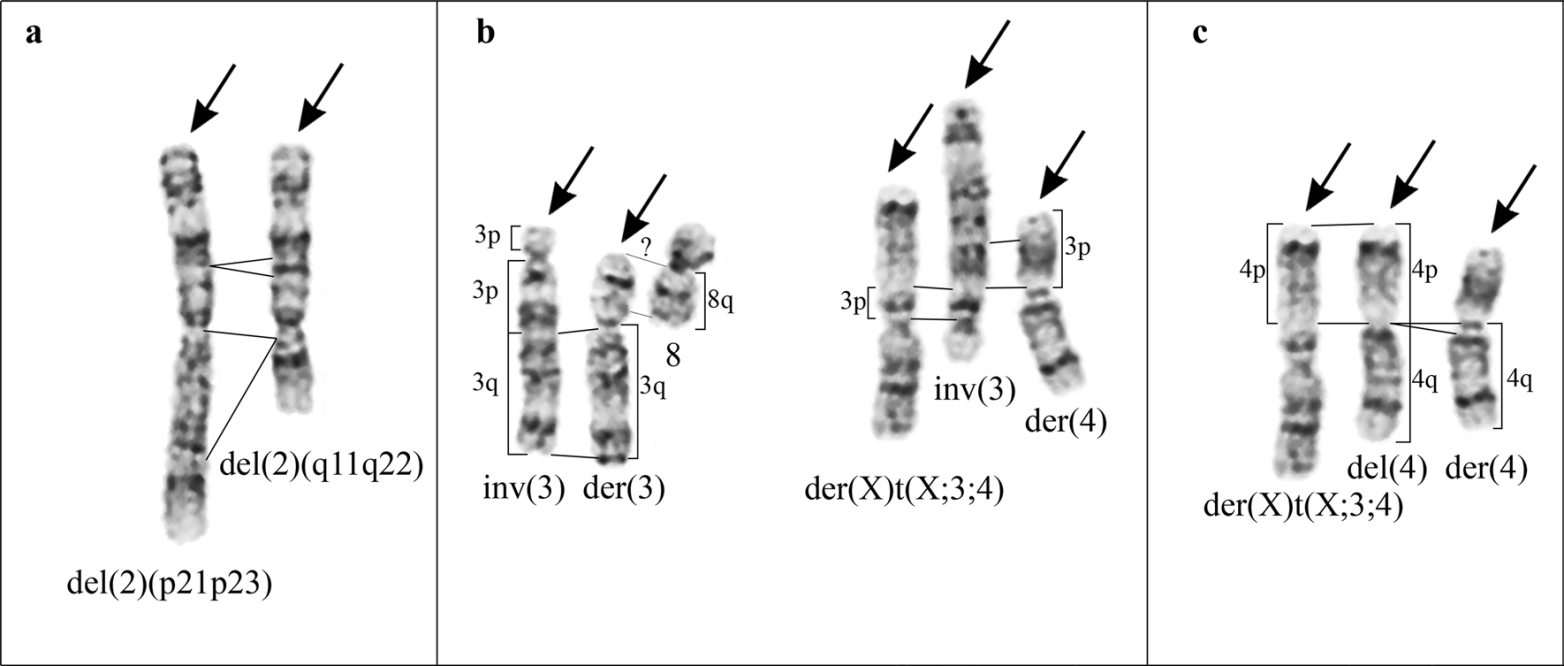
Identification of the 2nd, 3rd and 4th chromosome pairs. **a** Deletions of the short arm of the first chromosome 2 homologue, del(2)(p21p23), and of the long arm of the second chromosome 2 homologue, del(2)(q11q22). **b** Chromosome inv(3)(pter → p26::q11 → p26::q11 → qter) and distribution of the second chromosome 3 material between der(3), der(X), and der(4). **c** Chromosome del(4)(pter → q11::q12 → qter) and distribution of the second chromosome 4 material between der(X) and der(4). The arrows indicate structurally rearranged chromosomes. Deleted and corresponding chromosome regions marked by lines

Both chromosome 2 homologues have deletions (Fig. 2a). One homologue has an interstitial deletion del(2)(p21p23) of the short arm as a result of *DHFR* gene removal. Another homologue has extended deletion of the long arm, del(2)(q11q22). Chromosome inv(3)(p26q11) is an inverted chromosome 3 homologue (Fig. 2b). Chromosome der(3) contains the long arm of the second chromosome 3 homologue, while the material of its short arm is distributed between chromosomes der(X)t(X;3;4) and der(4). One chromosome 4 homologue (Fig. 2c) has a minor deletion del(4)(q11q12) in the pericentromeric region of the long arm. Another homologue is rearranged and fragmented. Its short arm is visible in chromosome der(X)t(X;3;4), whereas the long arm, possibly rearranged, is found in chromosome der(4). Chromosome add(6)(p11) consists almost entirely of one chromosome 6 homologue (Fig. 1). Another homologue is rearranged to a greater extent: 6q and Xq are likely combined in chromosome der(6). Chromosome add(8)(q11) contains the short arm of the second chromosome 8 homologue. Its long arm seems present in the short arm of chromosome der(3) (Fig. 2b). Complex rearrangements of the second chromosome 9 homologue and chromosomes 7 and 10 make their identification difficult. Probably the material of these chromosomes is distributed among marker chromosomes mar1—mar4.

**Fig. 3:**
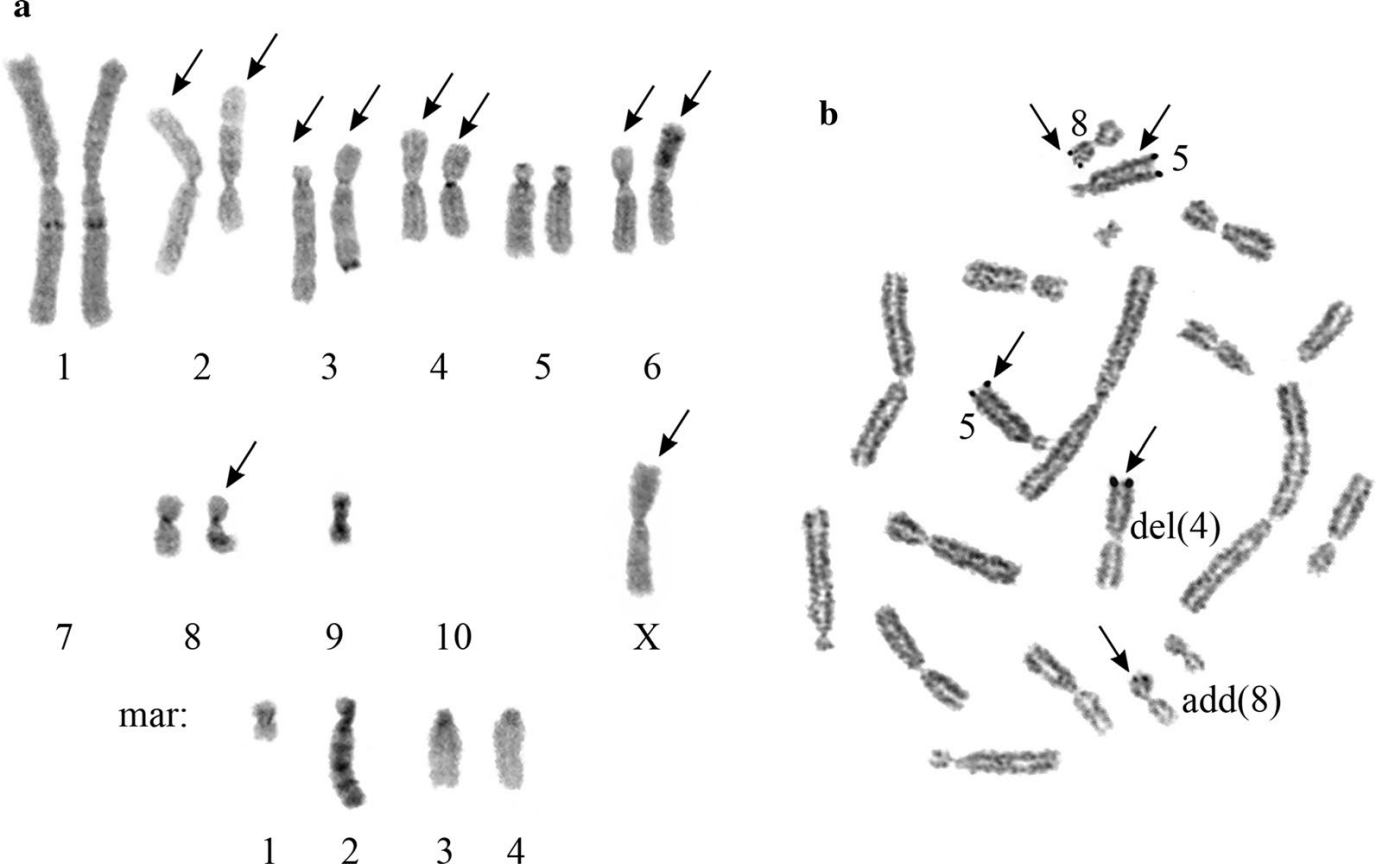
**a** C-banded karyotype of Chinese hamster ovary DXB-11 cell line (main SVK). The arrows indicate structurally rearranged chromosomes. **b** AgNOR staining pattern of DXB-11 chromosomes (metaphase spread). The arrows indicate Ag-positive chromosomes

C-bands were observed on the long arms of both chromosomes 1 and at the terminal region of the long arm of chromosome der(3) (Fig. 3a). In addition, the long arm of chromosome add(8)(q11) contained one C-positive region, whereas the mar2 long arm consisted of several repeated G- and C-positive regions varied in number in different cells (Figs. 1, 3a, 5a). The mutually exclusive rearrangements of chromosomes add(8)(q11) and mar2 observed in some cells allows to suggest that the heterochromatic material of the long arms of these chromosomes has the same origin and possibly belongs to chromosome 10 (Fig. 5b, c).

Five AgNORs in the DXB-11 cells (Fig. 3b) were found on the long arm of chromosome del(4)(q11q12), on the long arms of both chromosomes 5, and on the short arms of chromosomes 8 and add(8)(q11).

### Chromosomal instability

**Fig. 4:**
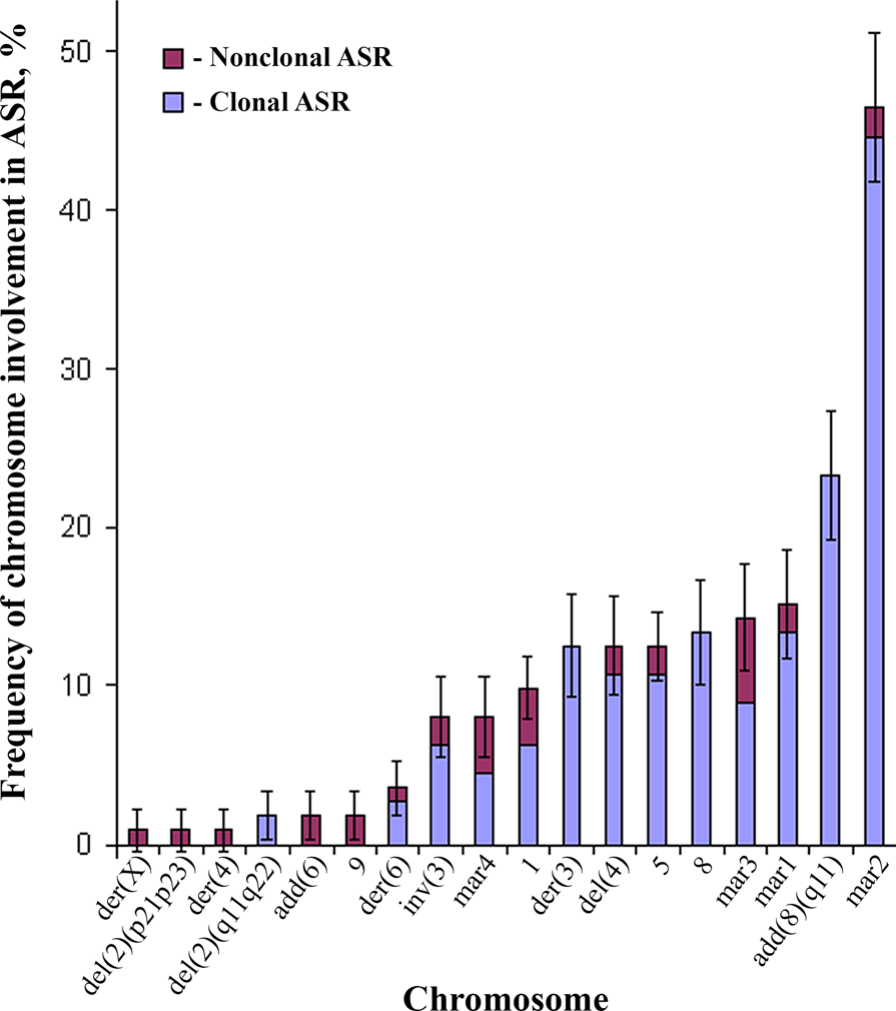
Differential instability of DXB-11 chromosomes. Horizontal axis—chromosomes of karyotype, vertical axis—frequency of chromosome involvement in ASR, %. Vertical bars are the errors of percentage

**Fig. 5:**
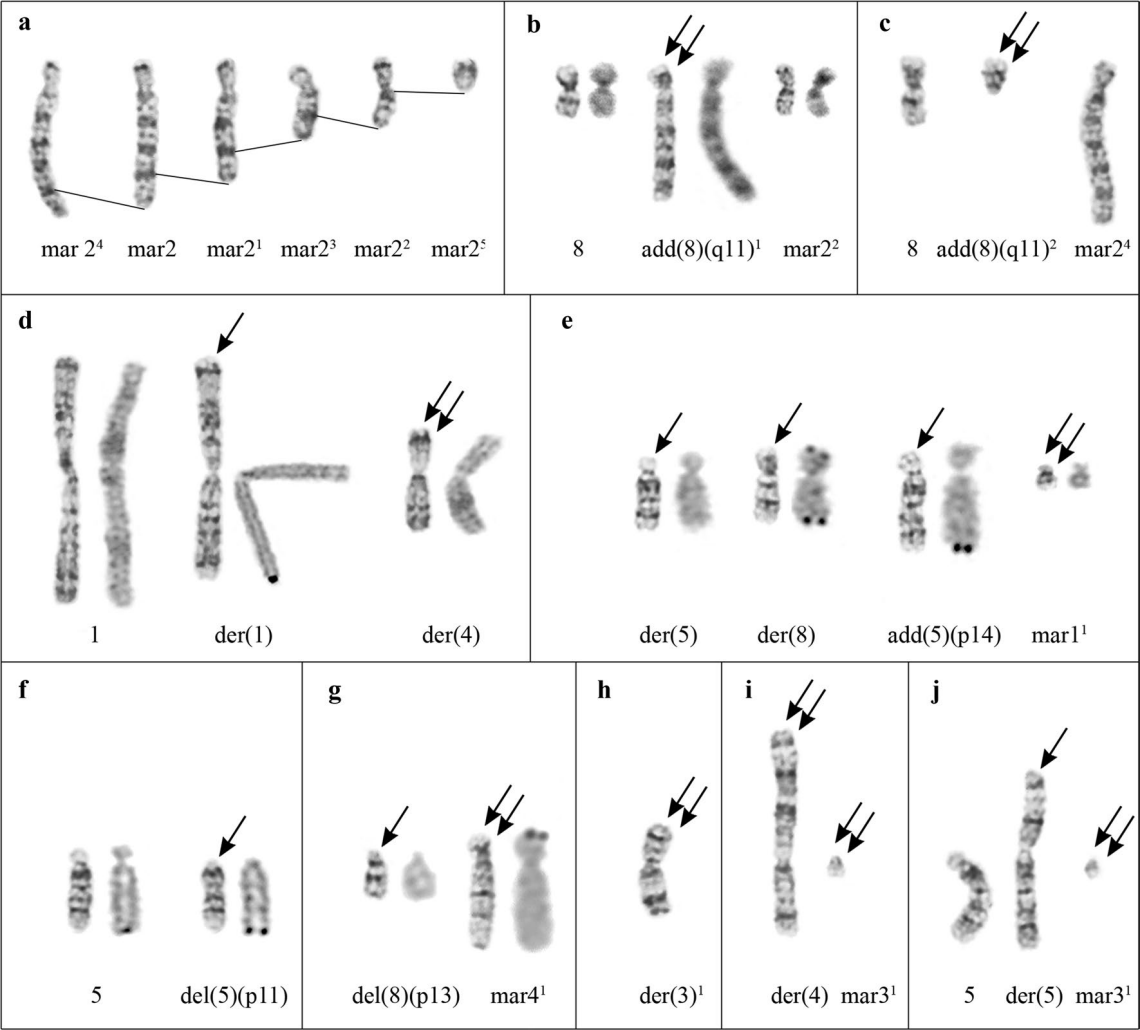
The most frequent clonal ASR. **a** Derivative variants of mar2 either with additional material on the long arm (mar2^4^) or with deletions of the long arm (shown by lines). **b** G- and C- banded chromosomes 8, add(8)(q11)^1^ and mar2^2^. **c** Deletion of the long arm of chromosome add(8)(q11), add(8)(q11)^2^ and mar2^4^. **d** der(1)(1pter → 1q42::4q26 → 4qter) and der(4)(4pter → 4q11::4q12 → 4q26::1q42 → 1qter) resulting from balanced translocation of chromosomes 1 and del(4)(q11q12), G- banding and Ag-staining. **e** der(5)(5pter → 5q28::8q25 → 8qter) and der(8)(8pter → 8q25::5q28 → 5qter) resulting from balanced translocation of chromosomes 5 and 8, and add(5)(?::p14 → qter) and deletion of the short arm of mar1, mar1^1^, G- banding and Ag-staining. **f** Chromosomes 5 and del(5)(p11), G- banding and Ag-staining. **g** del(8)(:p13 → pter) and derivative chromosome mar4^1^, G- banding and Ag-staining. **h** der(3)^1^ resulting from interstitial deletion of the long arm of chromosome der(3). **i, j** der(4)(?::4p33 → 4q11::4q12 → 4qter) and der(5)(?::5p12 → 5qter) resulting from the translocation of the long arm of mar3 to chromosomes del(4) (**i**) or 5 (**j**), and mar3^1^. Arrows indicate ASR of normal chromosomes of the main SVK. Double arrows indicate ASR of structurally rearranged chromosomes of the main SVK

**Fig. 6:**
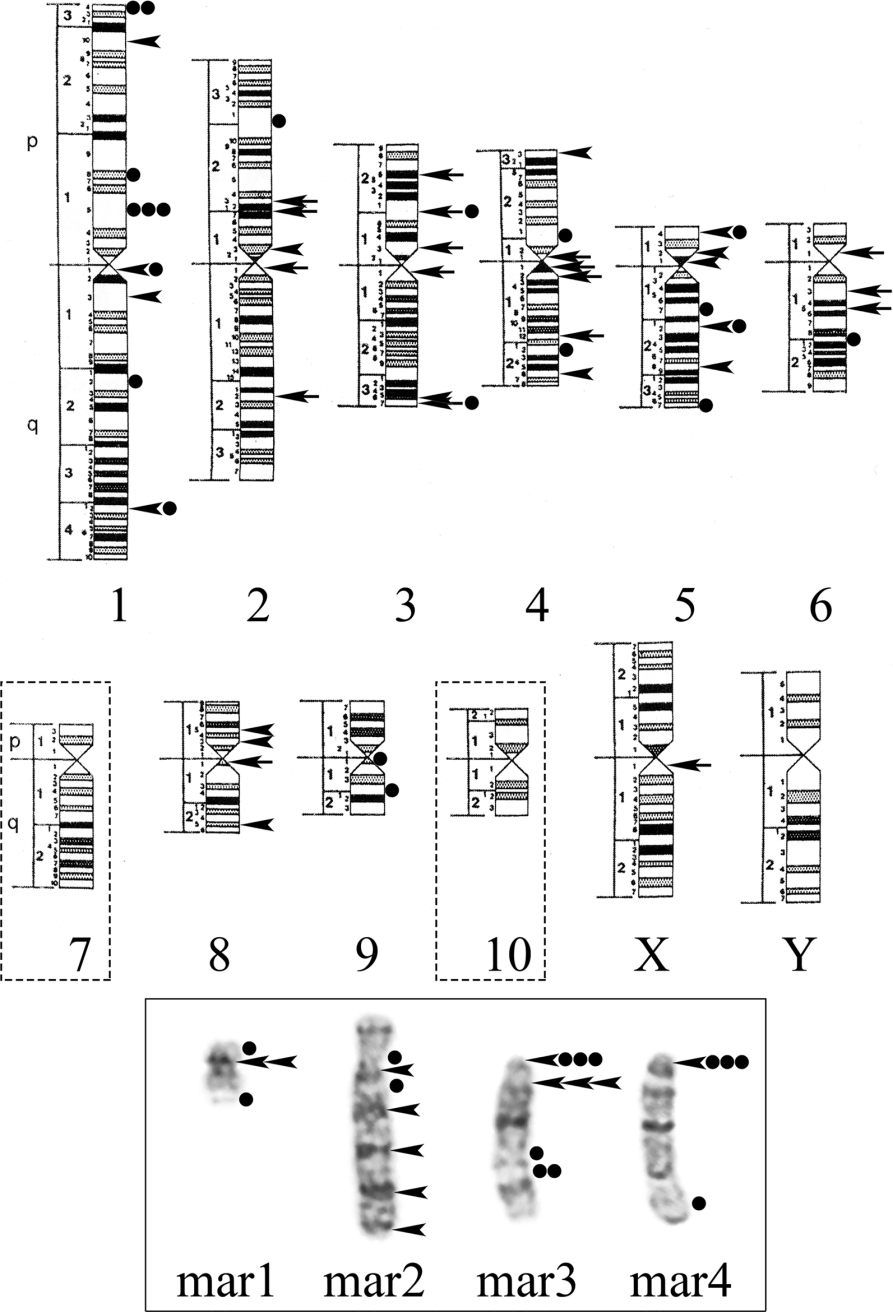
A schematic representation of chromosome loci involved in structural rearrangements in the main SVK (arrows), in clonal ASR (arrowheads) and in nonclonal ASR (circles) in DXB-11 cell line. In frame: images of the marker chromosomes of the main SVK. Idiograms of G-banding patterns for normal chromosomes of *Cricetulus griseus* [26] are used. In dotted frames: chromosomes 7 and 10 whose identification is impossible due to their complex rearrangements

All DXB-11 chromosomes participated in clonal and nonclonal ASR, although with different frequency (Figs. 4, 5, 6). The total number of chromosome involvements in ASR was 235, of which 197 (83.83%) were clonal and 38 were nonclonal (16.17%).

The most stable were chromosomes 9, der(X)t(X;3;4), del(2)(p21p23), del(2)(q11q22), der(4) and add(6)(p11), that were rarely involved in ASR (*p* < 0.05). Chromosome der(6) can also be considered a stable chromosome because its involvement in ASR was compatible with that of chromosomes inv(3)(p26q11) and mar4 (*p* > 0.05), but lower as compared to other chromosomes (*p* < 0.05). On the contrary, the most variable was chromosome mar2 (*p* < 0.05), which showed both extra copy and deletions of repeated regions of the long arm (Fig. 5a). Derivative variants of mar2 were marked by upper indexes according to the frequency of their occurrence. Thus, out of 112 cells, mar2 was revealed in 60 (53.57%), mar2^1^—in 22 (19.64%), mar2^2^—in 15 (13.39%), mar2^3^—in 6 (5.36%), mar2^4^—in 4 (3.57%), mar2^5^—in 3 (2.68%) cells. The rest of chromosomes had approximately the same degree of instability (*p* > 0.05). However, instability of chromosome add(8)(q11) was lower as compared to chromosome mar2 (*p* < 0.05), comparable with the instability of chromosome mar1 (*p* > 0.05), and higher (*p* < 0.05) as compared to all other chromosomes.

Inverse correlation of involvement of chromosomes add(8)(q11) and mar2 in ASR is of special interest (Fig. 5b, c). In 12 from 112 cells (10.71%), the long arm of chromosome add(8)(q11) contained additional G- and C-positive repeated regions, chromosome add(8)(q11)^1^ (Fig. 5b). These regions were similar, but did not coincide with the structure of the long arm of chromosome mar2, as shown by G-banding. Instead of chromosome mar2, a deleted one, chromosome mar2^2^, was found in these karyotypes (Fig. 5b). In one cell we also observed deletion of the long arm of chromosome add(8)(q11) which resulted in remaining of only the short arm, add(8)(q11)^2^, and appearance of chromosome mar2^4^ that had an extra G- and C-positive repeat on its long arm (Fig. 5c). Besides, deletion of chromosome add(8)(q11) long arm was detected in 6 from 112 cells (5.36%), regardless of structure of chromosome mar2 long arm.

Alternative pattern of involvement of other AgNOR chromosomes del(4)(q11q12), chromosomes of the 5th pair, and chromosome 8 in clonal ASR was revealed. So, for chromosome del(4)(q11q12), balanced translocation of chromosomes 1 and del(4)(q11q12) confirmed by Ag-staining (Fig. 5d) was the most frequent (9 from 112 cells, 8.04%). The balanced translocation of chromosomes 5 and 8, t(5;8)(q28;q25), was found in 8 from 112 cells (7.14%) (Fig. 5e). Also, rearrangement of the second chromosome 5 homologue was registered in these cells. Abnormal chromosome add(5)(p14) appears to be a result of translocation of the part of the short arm of chromosome mar1 to the chromosome 5 short arm (Fig. 5e). In addition, the deletion of the short arm of chromosome mar1 (mar1^1^) was registered in 7 from 112 cells (6.25%) without t(5;8)(q28;q25). Rare clonal ASR observed in 3 from 112 cells (2.68%) was the deletion of chromosome 5 short arm (Fig. 5f). Translocation of the short arm of chromosome 8 to the short arm of chromosome mar4 in 5 from 112 cells (4.46%) resulted in formation of two abnormal chromosomes, del(8)(p13) and mar4^1^ (Fig. 5g).

The most typical ASR of chromosome der(3) was the complex interstitial deletion of the long arm, der(3)^1^, observed in 11 from 112 cells (9.82%) (Fig. 5h). The clonal ASR of chromosome mar3 resulted in the appearance of small chromosome mar3^1^ containing mar3 pericentomeric region. The remaining part of the long arm of mar3 was translocated either to the chromosome del(4)(q11q12) short arm (Fig. 5i), or to the short arm of chromosome 5 (Fig. 5j), which was observed in 3 from 112 cells (2.68%) for every translocation.

As a whole, the chromosome material of different DXB-11 cells was similar in spite of their karyotype heterogeneity.

## Discussion

Like other CHO cell lines, DXB-11 cells were characterized by significant karyotypic heterogeneity. Apart from a small number of cells that had the same karyotype (main SVK), other variants of the DXB-11 karyotype were not identified. However, our analysis of ASR showed that individual chromosomes undergo rearrangements with different frequency. The revealed differential instability of DXB-11 chromosomes occurred to be in agreement with previous data [29] concerning different involvement of individual chromosomes in rearrangements in CHO cells (really, CHO-ori). This prompted us to perform a comparative karyotype analysis of some CHO cell lines for assessment of individual chromosome contribution to CHO karyotypic variability (Table 2).

**Table 2:** Comparison of the complement of normal and structurally rearranged chromosomes in CHO cell lines according to literary data

Chromosomes	CHO 21 < 2n > [16]	CHO 21 < 2n > [29]	CHO-K1 20 < 2n > [32]	CHO^dhfr−^ 20 < 2n > [20]	CHO-DUK 19 ~ 21 < 2n > [15]	DXB-11 20 < 2n > present work	DG-44 20 < 2n > [11]
X	+	+	Z1 and Z2/der(X)	der(X)t(X;4)	der(X)	der(X)t(X;3;4)	der(X)
	Z12	Z12	—	—	—	—	—
1	+	+	+	+	+	+	+
	Z1	+	+	del(1)(p15)	der(1)	+	+
2	+	+	+	+ ^a^	+ ^a^	del(2)(p21p23)	+
	Z2	Z2	Z3/Z2	del(2)/Z2	Z2	del(2)/Z2	Z2
3	Z3	Z3	Z4/der(Z3)	der(3)/der(Z3)	der(Z3)	der(3)/der(Z3)	mar1/der(Z3)
	Z4	Z4	Z9/Z4	inv(3)/Z4	Z4	inv(3)/Z4	Z4
4	Z5	Z5a	+ /Z5	+ /Z5	Z5	del(4)(q11q12)/Z5	+ /Z5
	Z7	Z7	Z6/Z7	mar1/Z7	Z7	der(4)/Z7	der(4)/Z7
5	+	+	+	+	+	+	+
	Z6	+	+	del(5)(p12)	der(5)	+	der(5)
6	Z8	Z8	Z5/Z8	add(6)/Z8	Z8	add(6)(p11)/Z8	Z8
	Z9	Z9	Z11/Z9	der(6)t(1;6)/der(Z9)	der(Z9)	der(6)t(X;?;6)/der(Z9)	der(6)/der(Z9)
7	+	+	+	—	—	—	—
	Z10	Z10	Z1/der(?Z10)	del(7)(p11)/?Z10	Z10	mar3/?Z10	der(7)/der(?Z10)
8	+	+	+	—	—	+	+
	Z11	+	Z8/der(Z11)	+ /der(Z11)	+ /der(Z11)	add(8)(q11)/der(Z11)	der(8)/der(Z11)
9	+	+	+	+ +	+	+	+
	Z13	Z13	Z12/Z13	del(9)/Z13	Z13	mar1/Z13	Z13
10	+	+	—	—	—	—	—
	—	—	—	—	—	—	der(10)
Marker chromosomes	—	—	Z10	—	—	mar2	mar2^b^
	—	—	—	mar2	mar1	—	—
	—	—	—	mar3	der(7)	mar4	—
	—	—	—	—	mar2	—	—
	—	—	—	—	mar3	—	—

The author’s designations of structurally rearranged chromosomes remain unchanged. After the slash, corresponding Z-chromosomes according to [16] or their derivatives are presented

+ normal chromosome homologue is present; — chromosome is absent

^a^ chromosome 2 has interstitial deletion of the short arm, judging by its image

^b^ the chromosome corresponds to mar2^1^ detected in DXB-11 cells in present work

According to the first description of karyotype of CHO cell line later named CHO-ori [2], the modal chromosome number was 21, and 9 routinely stained abnormal chromosomes were marked as “Z” [30]. Later, 8 normal (X, 1, 2, 5, 7, 8, 9, 10) and 13 structurally rearranged chromosomes (Z1—Z13) were identified in the CHO-ori karyotype by G-banding [16]. Subsequent analysis of CHO karyotype [29] did not reveal the rearrangements of the 1st, 5th and 8th chromosome pairs, but a minor change in the long arm of chromosome Z5 (Z5a) was documented. Other differences in the complement of normal and rearranged chromosomes, including their modal number, were not detected (Table 2). Thus, CHO-ori cells were aneuploid (2n = 22 in *Cricetulus griseus*) and were characterized by multiple structural rearrangements with partial loss of material of chromosomes X and 2.

Here, we have performed a comparative karyotype analysis of CHO derivatives (Table 2) in accordance with the specific nomenclature developed for Z-chromosomes [16] which is still being used despite a significant progress in identification of abnormal CHO chromosomes. Unfortunately, molecular hybridization studies using Bacterial Artificial Chromosome fluorescence in situ hybridization (BAC-FISH) [31] and chromosome painting probes [10, 13] were not accompanied by cell karyotyping.

A comparative analysis of CHO-ori, CHO-K1, CHO^dhfr−^, CHO-DUK, DXB-11 and DG-44 karyotypes demonstrated the presence of one normal chromosome homologue of the 1th, 5th and 9th pairs, as well as abnormal chromosomes Z2, Z4, Z5, Z7, Z8 and Z13. Apparently these chromosomes represent the most stable (i.e. conserved) part of CHO karyotype (Table 2).

Our data indicate that chromosomes 9, del(2)(p21p23), del(2)(q11q22) /Z2, der(4) /Z7, and add(6) /Z8 do undergo ASR in DXB-11 cells to a lesser extent. Stability of chromosomes 1, 2, 5, 8, 9, Z4, Z5, Z7 and Z8 in CHO-K1 and DG-44 cells was demonstrated by molecular cytogenetic methods [31]. Stability of chromosomes 1, 2, and 8 in DG-44 cells was shown by comparative genome hybridization [9]. CHO genome sequencing revealed the stability of chromosomes 1 and 4 /Z5 [17].

The variable part of CHO karyotype is represented by chromosomes X, 1 (2nd homologue), 5 (2nd homologue), 7, 8, 10, Z3, Z9, Z10, Z11, and Z12. Apparently rearrangements of these chromosomes determine genetic diversity and individuality of the karyotype structure of different CHO cell lines.

For example, establishment of CHO-K1 cell line was accompanied by a decrease in number of chromosomes from 21 (CHO-ori) to 20 (CHO-K1), and rearrangements of chromosomes X, 10, and abnormal chromosomes of the 3rd (Z3), 7th (Z10) and 8th (Z11) pairs [32, 33]. Chromosomes der(X)t(X;3;4), der(Z3), and der(Z11) found in CHO-K1 cells are also present in the karyotypes of DHFR-deficient cell lines CHO^dhfr−^, CHO-DUK, DXB-11 and DG-44. Chromosome der(Z11) contains an additional chromosome material on the long arm in DG-44 cells only [11]. We also found that chromosome add(8) /der(Z11) is often affected by rearrangements of its long arm.

Reorganization of material of chromosomes X (Xq), the 7th chromosome pair (7 and Z10), and chromosome 10 allows to distinguish CHO-K1 cells both from CHO-ori and CHO-K1 cells cultured in different conditions [10, 13, 31–33]. Rearrangements of the same chromosomes (Xq, 7 and 10) allow to distinguish DHFR-deficient CHO cell lines from CHO-K1 cells. Furthermore, the rearrangements of chromosomes Xq and 10 define peculiarities of karyotypes of DHFR-deficient CHO cells. According to our data, in DXB-11 cells chromosome mar2, probably der(10), is the most structurally variable and generally determines the karyotype heterogeneity of the cell population. DNA copy variations affect predominantly the same chromosomes, which are X, 7, 9/10, as well as chromosomes 5 and 6, as was shown by genome sequencing of different CHO cell lines [17].

Thus, different ability of DXB-11 chromosomes to undergo ASR is non-random and corresponds to individual chromosome instability in CHO cell lines. It has been suggested that some regions of the CHO genome are predisposed to structural variations [9]. Apparently, the stable chromosomes including those which are specific for various derivatives of CHO cells may be the preferred targets for transgene integration.

A significant difference between the cell lines DXB-11, CHO^dhfr−^ and CHO-DUK from CHO-K1 and DG-44 is a visible deletion in the short arm of chromosome 2, which is associated with the loss of *DHFR* gene at 2p23 [34]. The removal of both *DGFR* alleles in DG-44 cell line is not accompanied by a notable change of G-banding pattern of the short arm of chromosome 2 judging by karyotype image [11]. A deletion of the short arm of chromosome 2 allows distinguishing cell producer strains obtained on the basis of various host cells, namely either DXB-11 or CHO-DUK and DG-44.

We have shown here that the CHO cell line DXB-11 is not identical to CHO^dhfr−^ and CHO-DUK as was believed previously [12, 15, 20]. Despite the same modal number of chromosomes and the relative similarity of total chromosome material, CHO-DUK and DXB-11 cell lines have different karyotypes. In DXB-11 cells chromosomes of the 1st and 5th pairs and chromosome 8 are not rearranged. Besides, in these cell lines, chromosomes der(Z9) and mar2 are also different. The Z9 rearrangements are significant for differentiation of karyotypes of different CHO derivatives. Unlike CHO-ori and CHO-K1, in DHFR-deficient lines chromosome Z9 is rearranged. Derivative chromosome der(Z9) results from the translocation of chromosomes X and 6 in DXB-11 cells (present study) or chromosomes 1 and 6 in CHO^dhfr−^ [20] and CHO-DUK cells [12, 15]. It should be noted that two different chromosomes der(Z9) have similar abnormal structure of the long arm of chromosome 6. The chromosome der(Z9) in DG-44 cells has an unchanged long arm of chromosome 6 as revealed by G-banding [11]. However, molecular hybridization with BAC-FISH DNA probes [31] demonstrated that the DG-44 cells have an abnormal chromosome containing the material of chromosomes X and 6. In DXB-11 cells, chromosome mar2 differs from the chromosome referred to as mar2 in CHO^dhfr−^ [20] or, respectively, mar1 in CHO-DUK cells [12, 15] by the long arm structure. Interestingly, we have not found a single cell with the karyotype described for CHO-DUK/CHO^dhfr−^ cell lines despite the great karyotype diversity of DXB-11 cells.

Thus, it should be once again pointed out that the differences between DHFR-deficient cell lines DXB-11 and CHO-DUK/CHO^dhfr−^ are associated with chromosome rearrangements of the variable part of the CHO karyotype, namely, Xq, 1, 5, 8, 10, and Z9. It remains unclear whether DXB-11 and CHO-DUK cells are the result of the divergence of a single cell line cultured in different laboratories or these lines originate from the different experimentally obtained DHFR-deficient CHO cell clones [5]. According to Dr. Wurm’s concept, DXB-11 and CHO-DUK/CHO^dhfr−^ cell lines may be considered as CHO quasispecies [2].

The mechanisms of chromosomal/genomic instability of CHO cells remain poorly understood. According to our data, the breakpoints involved in formation of abnormal chromosomes in DXB-11 cells are often located in centromeric and pericentromeric regions (Fig. 6). It has been suggested that CHO chromosomal instability might be associated with telomeric repeats (TTAGGG)_n_ located in the pericentromeric regions of *Cricetulus griseus* chromosomes [31, 35]. At the same time, other types of DNA repeats including tandem repeats and transposable elements, such as endogenous retroviruses, long interspersed nuclear elements, short interspersed nuclear elements and DNA-transposons [36], might also contribute to the chromosomal instability of CHO cells. Analysis of the DXB-11 karyotype presented here may serve as a good basis for understanding the relationship between the localization of DNA repeats and chromosome breakpoints.

The genetic diversity of CHO cell lines appears at the level of both the karyotype and the genome. At the karyotype level, it shows through complex rearrangements which involve different chromosomes with different frequency (the differential instability), thus determining karyotype heterogeneity. At the genome level, sequencing of different CHO derivatives demonstrates single nucleotide polymorphisms, short insertions and deletions (InDels), DNA copy number variations and structural changes which lead to an increase in mutation frequency, the loss of genes and heterozygosity. Besides, different regions of the genome may undergo structural variations and genetic imbalance to a different extent [7, 8, 17]. Each CHO cell line is characterized by individual pattern of genomic changes and corresponding karyotype structure. However, data on the genomic variability of CHO cells obtained by different methods exist separately. Obviously, integrated approaches to study of karyotypic and genomic heterogeneity that would allow identifying relatively stable genome regions and regions that ensure its plasticity are necessary. Further exploration of the CHO cells phenomenon, understanding of the mechanisms of their genome plasticity might allow for more successful control the stability of recombinant cell lines.

## Conclusions

DXB-11 and CHO-DUK cell lines differ in karyotypes. DXB-11 cell population is characterized by a limited number of cells with identical chromosome complement and a predominant number of cells with a wide spectrum of clonal and nonclonal additional structural chromosome rearrangements. The revealed differential instability of DXB-11 chromosomes is most likely not incidental. Seemingly, karyotype heterogeneity of CHO cell lines is determined by rearrangements of variable CHO chromosomes.

## Data Availability

All data generated or analyzed during this study are included in this published article.

## Abbreviations

ASR: Additional structural rearrangements
AgNOR: Ag-positive nucleolar organizer region
SVK: Structural variant of karyotype
BAC-FISH: Bacterial artificial chromosome fluorescence in situ hybridization
